# Immunometabolic Analysis of *Mobiluncus mulieris* and *Eggerthella* sp. Reveals Novel Insights Into Their Pathogenic Contributions to the Hallmarks of Bacterial Vaginosis

**DOI:** 10.3389/fcimb.2021.759697

**Published:** 2021-12-23

**Authors:** Ross McKenzie, Jason D. Maarsingh, Paweł Łaniewski, Melissa M. Herbst-Kralovetz

**Affiliations:** ^1^ Department of Obstetrics and Gynecology, College of Medicine-Phoenix, University of Arizona, Phoenix, AZ, United States; ^2^ Department of Biology and Biochemistry, University of Bath, Bath, United Kingdom; ^3^ Department of Basic Medical Sciences, College of Medicine-Phoenix, University of Arizona, Phoenix, AZ, United States

**Keywords:** vaginal microbiome, vaginal dysbiosis, organotypic 3D culture, biogenic amines (BAs), global metabolic and regulatory networks, women’s health, cervical epithelial barrier, genital inflammation

## Abstract

The cervicovaginal microbiome plays an important role in protecting women from dysbiosis and infection caused by pathogenic microorganisms. In healthy reproductive-age women the cervicovaginal microbiome is predominantly colonized by protective *Lactobacillus* spp. The loss of these protective bacteria leads to colonization of the cervicovaginal microenvironment by pathogenic microorganisms resulting in dysbiosis and bacterial vaginosis (BV). *Mobiluncus mulieris* and *Eggerthella* sp. are two of the many anaerobes that can contribute to BV, a condition associated with multiple adverse obstetric and gynecological outcomes. *M. mulieris* has been linked to high Nugent scores (relating to BV morphotypes) and preterm birth (PTB), whilst some bacterial members of the *Eggerthellaceae* family are highly prevalent in BV, and identified in ~85-95% of cases. The functional impact of *M. mulieris* and *Eggerthella* sp. in BV is still poorly understood. To determine the individual immunometabolic contributions of *Eggerthella* sp. and *M. mulieris* within the cervicovaginal microenvironment, we utilized our well-characterized human three-dimensional (3-D) cervical epithelial cell model in combination with multiplex immunoassays and global untargeted metabolomics approaches to identify key immune mediators and metabolites related to *M. mulieris* and *Eggerthella* sp. infections. We found that infection with *M. mulieris* significantly elevated multiple proinflammatory markers (IL-6, IL-8, TNF-α and MCP-1) and altered metabolites related to energy metabolism (nicotinamide and succinate) and oxidative stress (cysteinylglycine, cysteinylglycine disulfide and 2-hydroxygluatrate). *Eggerthella* sp. infection significantly elevated multiple sphingolipids and glycerolipids related to epithelial barrier function, and biogenic amines (putrescine and cadaverine) associated with elevated vaginal pH, vaginal amine odor and vaginal discharge. Our study elucidated that *M. mulieris* elevated multiple proinflammatory markers relating to PTB and STI acquisition, as well as altered energy metabolism and oxidative stress, whilst *Eggerthella* sp. upregulated multiple biogenic amines associated with the clinical diagnostic criteria of BV. Future studies are needed to evaluate how these bacteria interact with other BV-associated bacteria within the cervicovaginal microenvironment.

## Introduction

In healthy reproductive age women, the cervicovaginal microbiome is generally dominated by *Lactobacillus* spp. These beneficial bacteria acidify the cervicovaginal microenvironment *via* lactic acid production, which contributes to protection against infections by pathogenic and opportunistic microorganisms (O’hanlon et al., 2011). The depletion of *Lactobacillus* spp. leads to the colonization of the lower female reproductive tract (FRT) by a diverse consortium of facultative and obligate anaerobic bacteria, a disorder is referred to as bacterial vaginosis (BV) (Workowski and Bolan, 2015; Muzny et al., 2020). Importantly, BV is associated with a range of adverse gynecologic and obstetric outcomes including an increased risk of sexually transmitted infections (STI) and preterm birth (PTB). Microbiologically, BV is characterized by the presence of a polymicrobial biofilm covering the surface of cervicovaginal epithelium (Muzny et al., 2019). *Mobiluncus mulieris* and *Eggerthella* sp. are two of the many anaerobes that may contribute to the biofilm formation, yet their mechanistic contributions to BV and related adverse gynecologic and obstetric outcomes are still poorly understood (Danielsson et al., 2011; Machado and Cerca, 2015).

*Mobiluncus* spp. are motile, curved rod-shaped bacteria that are isolated from vaginal secretions from women with BV and are associated with high Nugent scores (a method to diagnose BV) (Sprott et al., 1983; Roberts et al., 1985; Hallén et al., 1987; Teo et al., 1987; Vetere et al., 1987; Moi et al., 1991; Gatti, 2000; Srinivasan and Fredricks, 2008). In addition, *M. mulieris* has been isolated from extragenital sites, such as breast and umbilical abscesses (Glupczynski et al., 1984). In previous epidemiological studies, *M. mulieris* have been also linked to PTB (Holst et al., 1994; Hillier et al., 1995; Meis et al., 1995). Genital inflammation has been implicated in PTB and *M. mulieris* has been hypothesized as a microbial driver in such inflammatory states (Dude et al., 2020). The flagella of *M. mulieris* has been previously demonstrated to stimulate Toll-like receptor 5 (TLR5) activation, which links to the elevation of key inflammatory markers (IL-6, IL-8, and TNF-α) and PTB (Anahtar et al., 2015; Onderdonk et al., 2016; Dude et al., 2020; Dela Cruz et al., 2021). In addition, *M. mulieris* has been shown to exert sialidase activity (Culhane et al., 2006). Notably, this bacterial enzyme cleaves sialic acid from highly glycosylated proteins present in the cervical mucus plug and its activity is associated with BV (Smayevsky et al., 2001), PTB and chorioamnionitis (Critchfield et al., 2013; Racicot et al., 2013; Smith-Dupont et al., 2017).

Some bacterial members of the *Eggerthellaceae* family are highly prevalent in BV, identified in ~85-95% of cases (Fredricks et al., 2005; Fredricks et al., 2007; Srinivasan et al., 2012; Shipitsyna et al., 2013). Interestingly, one member of the *Eggerthellaceae* family have also been linked to all four of the Amsel criteria (vaginal pH, vaginal odor, vaginal discharge and the presence of clue cells) used to diagnose BV in clinical settings (Srinivasan et al., 2015). *Eggerthella* spp. [previously classified as *Eubacterium* (Kageyama et al., 1999)] are non-motile anaerobic coccobacilli that are part of the healthy human gut microbiome (Finegold et al., 1983; Schwiertz et al., 2000). The taxonomy of the *Eggerthellaceae* family requires further investigation to classify them into their appropriate genus and species. However, *Eggerthella* spp. can also cause bacteremia and sepsis with high mortality rates (Lau et al., 2004a; Lau et al., 2004b; Thota et al., 2011; Lee et al., 2012). This suggests that in the FRT, *Eggerthella* spp. might play a role in the pathophysiological processes that manifest as adverse obstetric and gynecologic outcomes.

To determine the individual immunometabolic contributions of *Eggerthella* sp. and *M. mulieris* within the cervical microenvironment, we utilized our well-characterized human three-dimensional (3-D) cervical epithelial cell model that recapitulates several physiologically relevant features of *in vivo* tissue, including TLR expression, microvilli, intercellular junctional complexes and secretory material. We combined this advanced bioreactor-derived 3-D cell culture model with multiplex immunoassay and global untargeted metabolomics approaches to identify key immune mediators and metabolites related to *M. mulieris* and *Eggerthella* sp. infections of the lower FRT. We chose the 3-D cervical model since the cervix is a critical area impacted by cervicovaginal microbiota that, when disrupted, can lead to PTB, increased STI acquisition and other gynecological sequalae associated with BV.

## Methods

### Human Cervical Epithelial Cell Culture and Generation of the 3-D Cervical Model

Human cervical epithelial cells (A2EN) were generously provided by Dr. Alison Quayle at Louisiana State University Health Sciences Center (Herbst-Kralovetz et al., 2008; Buckner et al., 2011) and were routinely maintained in keratinocyte serum-free media (KSFM) (Fisher Scientific) supplemented with epidermal growth factor (5 ng/ml), bovine pituitary extract (50 µg/ml), CaCl_2_ (Gibco) and primocin (100 µg/ml; *In vivo*Gen) at 37°C in a 5% carbon dioxide (CO_2_) humidified atmosphere. Short tandem repeat DNA profiling confirmed that cells were not contaminated with other cell lines found in available databases. For downstream experiments, we used cervical epithelial cells (passage ~50–60) cultured as monolayers or 3-D cervical cell models. Monolayer cultures were seeded at ~2 × 10^5^ cells/ml into tissue culture-treated 24-well plates. Prior to seeding, cells were enumerated by trypan blue exclusion. The 3-D cervical cell models were generated as previously described (Radtke and Herbst-Kralovetz, 2012; Radtke et al., 2012; Jackson et al., 2020). Briefly, cervical epithelial cell monolayers were trypsinized and counted using a Countess automated cell counter (Invitrogen). The single cell suspension (~1 × 10^7^) was combined with 300 mg of hydrated Cytodex-3 collagen-coated dextran microcarrier beads (Sigma-Aldrich) suspended in pre-warmed KFSM-primocin medium. The mixture was transferred to a rotating-wall vessel (RWV) bioreactor (Synthecon). Bioreactors were incubated at 37°C for 28-days at 20 rpm, with daily medium changes. After 28-days the 3-D cervical cell models were harvested, washed and resuspended in antibiotic-free KFSM medium, enumerated, and distributed into 24-well plates at a density of ~5 x 10^5^ cells/well for downstream experiments.

### Bacterial Strains and Growth Conditions

All bacterial strains used in this study were obtained from the Biodefense and Emerging Infections (BEI) Research Repository (NIAID, NIH as a part of the Human Microbiome Project). *M. mulieris* strain UPII-28I and *Eggerthella* sp. strain MVA1 were cultured on tryptic soy agar (TSA) (Becton Dickinson) supplemented with 5% defibrinated sheep blood (Quad Five) at 37°C under anaerobic conditions generated using anaerobic environment chambers and AnaeroPacks (Thermo Scientific). Due to large taxonomic restructuring of vaginal species over the last five years we decided to confirm our strains taxonomic classification. Although not much genomic information is available yet for *Eggerthella* sp. strain MVA1 there is a sequence read SRX655730 in the NCBI Sequence Read Archive. This read in the SRA reports that *Eggerthella* sp. MVA1 has 86.46% sequence identity with the *Eggerthellaceae* family and 83.23% identity with the *Eggerthella* genus using their Sequence Taxonomic Analysis Tool (STAT) (Katz et al., 2021). There is also a 16S rRNA sequence (JX103988) available that has 99% sequence identity with *Eggerthella lenta*. Future comparative genomic analyses are needed to designate a species for *Eggerthella* sp. strain MVA1.

### Bacterial Infections

*M. mulieris* UPII-28I and *Eggerthella* sp. MVA1 were cultured on TSA agar with sheep’s blood for 16-18 hours prior to infection. Bacterial strains were harvested and resuspended in sterile Dulbecco’s phosphate-buffered saline (PBS) and adjusted to an optical density at 600 nm (OD_600_) for infection assays. The OD_600_ 0.5 reflected the CFU/ml range of 1 x 10^8^ – 1 x 10^9^, likely due to bacterial cell clumping as observed on the SEM. Monolayers were infected with adjusted bacterial suspensions (20 μl of bacterial suspension adjusted to OD_600_ of 0.05, 0.5 and 5.0 per 1 x 10^5^ cells and incubated for 24 hours under anaerobic conditions at 37°C for use in cytotoxicity assays. The 3-D cervical cell aggregates were infected with adjusted bacterial suspensions (20 μl of bacterial suspension adjusted to OD_600_ of 0.5 per 1 x 10^5^ cells and incubated under anaerobic conditions at 37°C for 24-hours. In a preliminary experiment the bacterial recovery 24 hours after the infection of the 3-D cervical cell model with both *M. mulieris* UPII-28I and *Eggerthella* sp. MVA1 was within 0.5 of a log of the initial infection dose. PBS-treated cells served as mock-infected controls. Culture supernatants were immediately used for cytotoxicity assays or stored at -80°C for downstream immunoproteomic and metabolomic analyses.

### Lactate Dehydrogenase Assay (LDH)

Culture supernatants from cervical epithelial monolayer cell infections were used to assess cytotoxicity using the CyQUANT LDH assay (Thermo Fisher Scientific) according to the manufacturer’s protocol. LDH activity was measured by recording absorbance values at 490 nm and 680 nm and the percentage LDH activity was calculated according to the equation: $\frac{\text{sample LDH activity}}{\text{lysed control LDH activity}}\times 100.$ The assay was performed using three independent biological replicates.

### Scanning Electron Microscopy

Human 3-D cervical cell models were infected with *M. mulieris* UPII-28I and *Eggerthella* sp. MVA1 for four hours under anaerobic conditions at 37°C. Samples were fixed in 2.5% glutaraldehyde (Electron Microscopy Sciences) and prepared for scanning electron microscopy (SEM) as described previously (Hjelm et al., 2010; Mcgowin et al., 2013). Infected 3-D cervical cell aggregates were imaged with a JSM-6300 JEOL scanning electron microscope and IXRF model 500 digital processor (IXRF systems) at the Electron Microscopy Core at Arizona State University. Representative images collected for each bacterium were selected for inclusion in the figure. Pseudo-coloring of the SEM images was performed using Adobe Photoshop CS6 v13.

### Multiplex Immunoassays

Cell culture supernatants from 3-D cervical cell models infected with *M. mulieris* UPII-28I and *Eggerthella* sp. MVA1 were collected from three independent experiments. The levels of five cytokines: (interleukin (IL)-1α, IL-1β, IL-1RA, IL-6, tumor necrosis factor-α (TNF)-α), seven chemokines: fractalkine, IL-8, interferon γ-induced protein-10 (IP-10), monocyte chemoattractant protein (MCP)-1, MCP-3, macrophage inflammatory protein-1β (MIP-1β), regulation on activation, normal T-cell expressed and secreted (RANTES) and three growth factors: platelet derived growth factor-AA (PDGF-AA), transforming growth factor-α (TGF-α), vascular endothelial growth factor (VEGF) were measured using customized MILLIPLEX^®^ multianalyte profiling (MAP) Human Cytokine/Chemokine Panel 1 array (Millipore) and compared to PBS mock infections. Data was collected using a Bio-Plex® 200 (Bio-Rad) platform and evaluated using Manager (5.0) software (Bio-Rad). A five-parameter logistic regression curve fit was used to determine the concentration. All samples were analyzed in biological triplicate, each containing two technical replicates.

### Untargeted Metabolomics Analysis

Cell culture supernatants from 3-D cervical cell models infected with *M. mulieris* UPII-28I and *Eggerthella* sp. MVA1 from three independent experiments were sent to Metabolon Inc. (Durham, NC) for untargeted global metabolomics analysis. Metabolites were resolved using ultra-performance liquid chromatography with mass spectrometry (UPLC-MS) as described previously (Ilhan et al., 2020; Salliss et al., 2021). The sample extracts were dried then reconstituted in solvents compatible to four different methods. Sample aliquots were analyzed using: acidic positive ion conditions that were chromatographically optimized for more hydrophilic or hydrophobic compounds, basic negative ion optimized conditions and negative ionization conditions. The MS analysis used dynamic exclusion with a scan range covering 70-1000 m/z. The Laboratory Information Management System (LIMS) was used for data extraction and peak-identification, QC and compound identification.

### Statistical Analysis

All assays and infections were performed as at least three biological replicates. Statistical differences between the mean protein concentrations among groups were determined by one-way ANOVA with Bonferroni *post-hoc* test using Prism v9.1.1 software (GraphPad). ClustVis (Metsalu and Vilo, 2015) was used to perform hierarchical clustering analysis (HCA) on the Bio-Plex data (ln-transformed and Pareto scaled, Euclidean distance measures and average linkage clustering). Metabolomics data analyses, including HCA, Spearman’s correlation analysis, principal component analysis (PCA) and metabolite enrichment pathway analysis, were performed with MetaboAnalyst 5.0 (Pang et al., 2021). Prior to analysis the metabolomics data was log-transformed, and Pareto scaled. Relative abundance is the normalized values from the area under the curve of the metabolite peaks collected that are rescaled to set the median equal to 1, before inputting any missing values as the minimum. To determine the significance between the mean relative abundances of metabolites among groups (infection vs. PBS control), two-tailed paired Student’s t-tests was performed using the rstatix R package. To correct for multiple comparisons, *p*-values were adjusted using false discovery rate (FDR) and *q*-values were reported. *p*-values below 0.05 were considered significant.

## Results

### *Eggerthella* sp. and *Mobiluncus mulieris* Do Not Induce Significant Cytotoxicity in Colonized 3-D Cervical Epithelial Cell Models

First, we assessed whether *Eggerthella* sp. and *M. mulieris* infections induced cytotoxicity in cervical epithelial cell monolayers at three doses which corresponded with the final OD_600_ of 0.1, 0.01 and 0.001 of 1x10^5^ cervical cells/ml. Using LDH cytotoxicity assays, we found that there was no significant cytotoxicity induced following infection with *Eggerthella* sp. and *M. mulieris* at any dose tested (**Supplementary Figure 1**).

**Figure 1:**
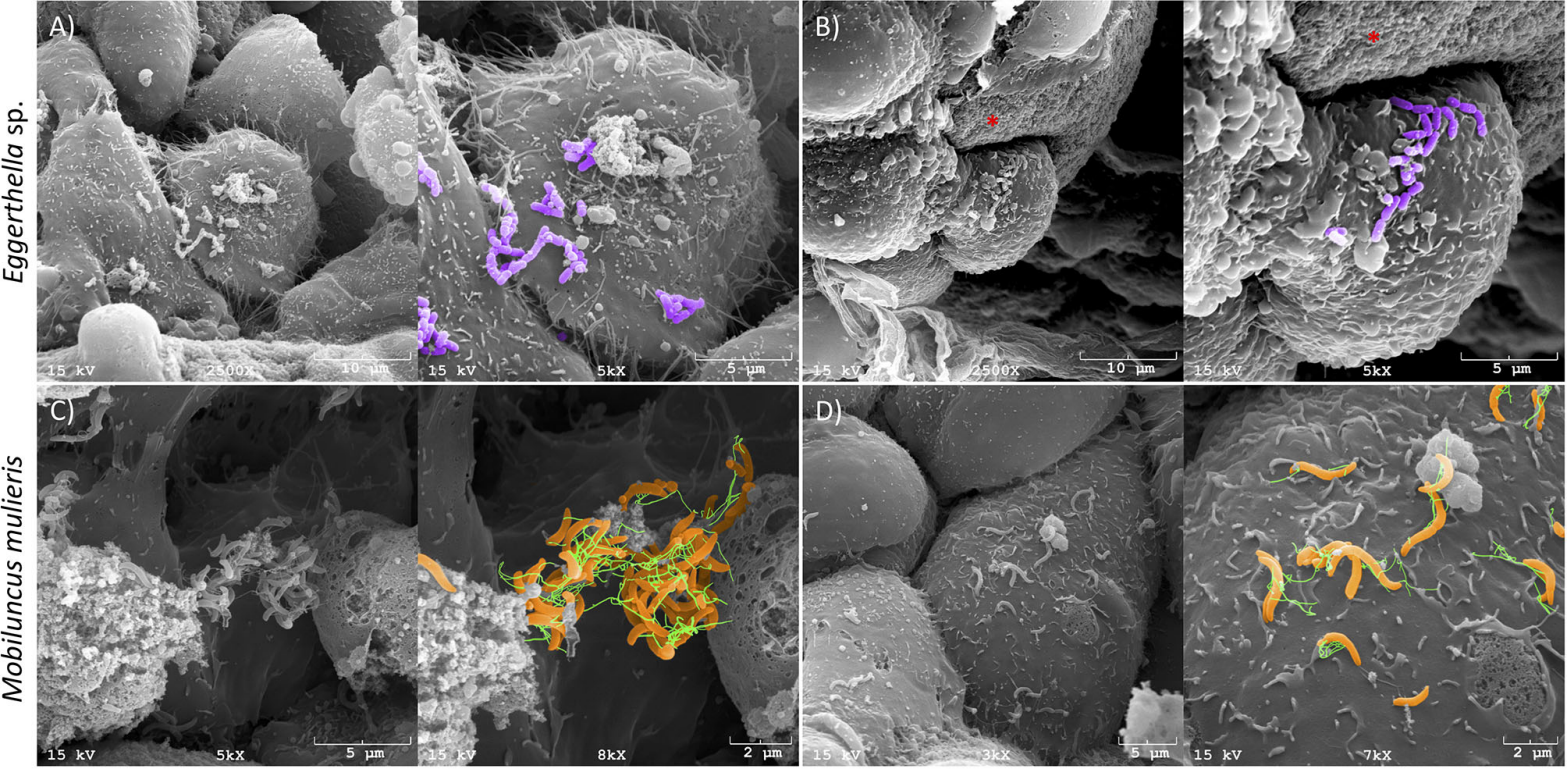
*Eggerthella* sp. and *M. mulieris* colonize 3-D human cervical epithelial cell models. Pseudo-colored scanning electron microscopy (SEM) images of **(A)** and **(B)**, *Eggerthella* sp. MVA1 and **(C, D)**, *M. mulieris* UPII-28I showing colonization of 3-D cervical epithelial cells. *Eggerthella* sp. MVA1 exhibit a coccobacilli morphology and colonized 3-D cervical cells in small clusters or short chains. *M. mulieris* UPII-28I cells exhibit a curved rod-shaped morphology and were pseudo-colored orange with flagella-like structures pseudo-colored green. The * indicates the collagen-coated microcarrier beads used to generate the 3-D cervical epithelial cell models.

We confirmed colonization of 3-D cervical cell models with *Eggerthella* sp. and *M. mulieris* by SEM (**Figure 1**). Both *Eggerthella* sp. and *M. mulieris* formed clusters and interacted simultaneously with multiple cells in some areas. *Eggerthella* sp. colonized the 3-D cervical cell models in smaller clusters and longer chains (**Figures 1A, B**). *M. mulieris* exhibited flagella-like structures which appeared to interact with other bacterial cells (**Figure 1C**) and epithelial cell surfaces (**Figure 1D**).

### Infection of 3-D Cervical Aggregates With *M. mulieris* Upregulated Levels of Several Key Proinflammatory Cytokines and Chemokines, Whereas Infection With *Eggerthella* sp. Elevated IL-1α Secretion

To investigate the host immune response to *M. mulieris* and *Eggerthella* sp., we infected 3-D cervical cell models with each bacterium for 24 hours and measured levels of secreted cytokines (IL-1α, IL-1β, IL-1RA, IL-6, TNF-α), chemokines (fractalkine, IL-8, IP-10, MCP-1, MCP-3, MIP-1β, RANTES) and growth factors (PDGF-AA, TGF-α, VEGF). Data from the infectious conditions were compared to PBS mock-infected controls.

**Figure 2:**
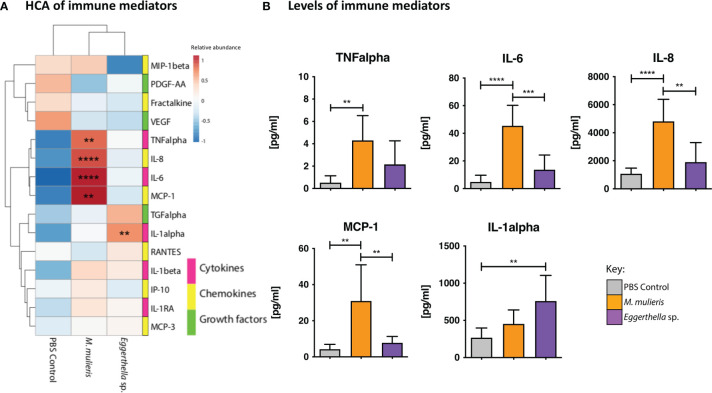
*M. mulieris* significantly elevated production of inflammatory cytokines and chemokines compared to *Eggerthella* sp. and PBS mock-infected controls in 3-D cervical aggregates. **(A)** Hierarchical clustering analysis (HCA) of cytokine, chemokine, and growth factor profiles secreted by 3-D cervical cells in response to infection with *Eggerthella* sp. MVA1 or *M. mulieris* UPII-28I. The data was log-transformed and Pareto-scaled prior to clustering. HCA was performed using Euclidean distance measures and average linkage clustering algorithms. **(B)** Bio-Plex analysis of cytokines, chemokines and growth factors secreted by 3-D human cervical cells infected with *M. mulieris* UPII-28I and *Eggerthella* sp. MVA1 for 24h in anaerobic conditions. TNF-α, IL-6, IL-8 and MCP-1 were all significantly elevated by *M. mulieris* UPII-28I, whilst *Eggerthella* sp. MVA1 significantly increased expression of IL-1α. Statistical significance was determined by one-way ANOVA and Bonferroni *post-hoc* multiple comparisons. **, *p*<0.01; ***, p<0.001; ****, *p*<0.0001.

We performed hierarchical clustering analysis (HCA) to visualize patterns of immune mediator expression by 3-D cervical cell models in response to bacterial infection (**Figure 2A**). HCA demonstrated distinct immune mediator profiles of *M. mulieris* and *Eggerthella* sp. as each condition clustered separately from the PBS mock-infected controls. Using the multiplex assays, we found that infection of 3-D cervical cell models with *M. mulieris* significantly upregulated expression of IL-6 (*p*<0.0001), IL-8 (*p*<0.0001), MCP-1 (*p*<0.01) and TNF-α (*p*<0.01) whereas infection with *Eggerthella* sp. significantly upregulated only IL-1α (*p*=0.01) (**Figure 2B** and **Supplementary Figure 2**). IL-6, IL-8, TNF-α and IL-1α are all proteins linked to increased genital inflammation (Hannun and Obeid, 2018; Łaniewski et al., 2018). This data indicated that *M. mulieris* promoted a proinflammatory response in 3-D cervical cell models to a greater extent than *Eggerthella* sp.

### *Eggerthella* sp. and *M. mulieris* Infections Distinctly Altered Extracellular Metabolomes Corresponding to Amino Acid and Lipid Superpathways in 3-D Cervical Epithelial Cell Models

**Figure 3:**
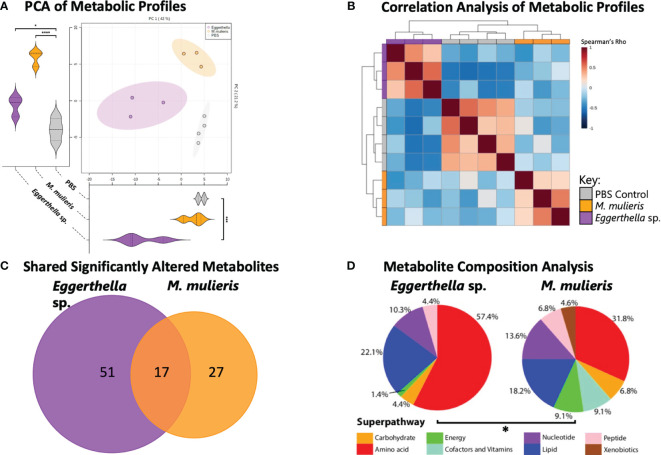
*M. mulieris* and *Eggerthella* sp. infections of 3-D cervical cell models resulted in distinct metabolic profiles. **(A)** Principal component analysis (PCA) shows distinct clustering between each bacterial metabolic profiles and the PBS mock-infected controls. PC1 and PC2 score significance was determined using one-way ANOVA with Bonferroni *post-hoc* tests. *, *p*<0.05; ***, p<0.001; ****, *p*<0.0001. **(B)** Spearman’s correlation heatmap of metabolic profiles demonstrating clustering of biological replicates for each infection. **(C)** Venn diagram indicating the unique or overlapping metabolites that were significantly altered (*p*<0.05) between the two bacterial infections. The significant differences in metabolite abundances among infections were determined using Student’s t-tests with Welch’s correction and compared to PBS mock-infected controls. **(D)** Pie charts showing the percentage of significantly (*p*<0.05) altered metabolites grouped by superpathway compared to PBS mock-infected controls (total number of significantly changed metabolites for *Eggerthella* sp. MVA1 and *M. mulieris* UPII-28I were 68 and 44 respectively). The significant difference between composition of superpathways was determined with chi-squared (χ^2^) test (*, *p*<0.05).

To discern the effect of *Eggerthella* sp. and *M. mulieris* infections on the cervicovaginal extracellular metabolome, we performed untargeted global metabolomics analysis using supernatants collected from 3-D model experiments. The metabolomics analysis identified 314 known metabolites. To compare global metabolic profiles of *Eggerthella* sp. and *M. mulieris*, principal component analysis (PCA) and Spearman’s correlation analysis (**Figure 3**) were employed. Biological replicates from each bacterial infection and PBS mock-infected controls clustered together and showed distinct separation of each condition by PCA (**Figure 3A**). Principal component 1 (PC1) explained 42% of variance and was significantly different (*p<*0.001) between *Eggerthella* sp. and mock-infected controls; principal component 2 (PC2) explained 21.2% of the variance scores and contributed to separation of *M. mulieris* and *Eggerthella* sp. from the mock-infected controls (*p*<0.05). Spearman’s correlation analysis showed each bacterial infection and the PBS mock-infected controls clustered distinctly from one another with each of the biological replicates grouped together (**Figure 3B**), therefore showing good replicability, and supporting the PCA analysis.

Overall, infection with *Eggerthella* sp. and *M. mulieris* significantly (*p*<0.05) altered the abundance of 68 and 44 metabolites, respectively, compared to mock-infected controls (**Supplementary Figure 3**). Of these differentially abundant metabolites, *Eggerthella* sp. and *M. mulieris* shared 17 significantly altered metabolites (**Figure 3C**). Next, we grouped significantly altered metabolites by superpathway and compared superpathway profiles between the two bacterial infections. Metabolites representing the amino acid superpathway (57.4% and 31.8% respectively) and the lipid superpathway (22.1% and 18.2% respectively) were profoundly influenced by infection with *Eggerthella* sp. and *M. mulieris* (**Figure 3D**). The overall composition of the superpathways between *Eggerthella* sp. and *M. mulieris* was significantly different (*p*=0.0147).

**Figure 4:**
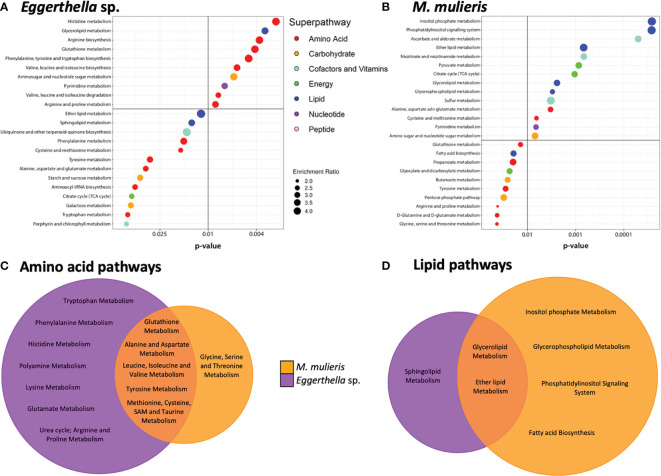
*Eggerthella* sp. primarily altered amino acid subpathways whilst *M. mulieris* significantly altered lipid-related subpathways. Metabolic pathway enrichment analysis for **(A)** *Eggerthella* sp. MVA1 and **(B)** *M. mulieris* UPII-28I infections of 3-D cervical cell models. All subpathways shown were significantly enriched (*p*<0.05) using metabolite set enrichment analysis (MSEA). Colored circles next to the subpathways indicate which superpathway each subpathway belongs to. Venn diagrams comparing the significantly altered (*p*<0.05) **(C)** amino acid and **(D)** lipid subpathways by *Eggerthella* sp. MVA1 and *M. mulieris* UPII-28I infections.

Next, we conducted metabolic pathway enrichment analysis on the metabolomics data sets to identify metabolic pathways significantly enriched by each bacterial infection (**Figure 4**). *Eggerthella* sp. infection significantly (*p*<0.05) enriched 23 subpathways, mostly associated with the amino acid superpathway (**Figure 4A**) while *M. mulieris* infection significantly enriched 24 subpathways and the most significant were from the lipid superpathway (**Figure 4B**). We also compared and contrasted these subpathways between *Eggerthella* sp. and *M. mulieris* (**Figures 4C, D**). Following comparisons of amino acid and lipid subpathways, we observed that *Eggerthella* sp. enriched a vast number of amino acid subpathways, twice that of *M. mulieris.* The majority of subpathways enriched by *M. mulieris* were also enriched by *Eggerthella* sp. Conversely, we noted that *M. mulieris* enriched twice the number of lipid subpathways than *Eggerthella* sp., with only sphingolipid metabolism being unique to *Eggerthella* sp. infection.

### *Eggerthella* sp. Infection Significantly Altered Levels of Sphingolipids and *M. mulieris* Infections Significantly Altered Levels of Long-Chain Fatty Acids

**Figure 5:**
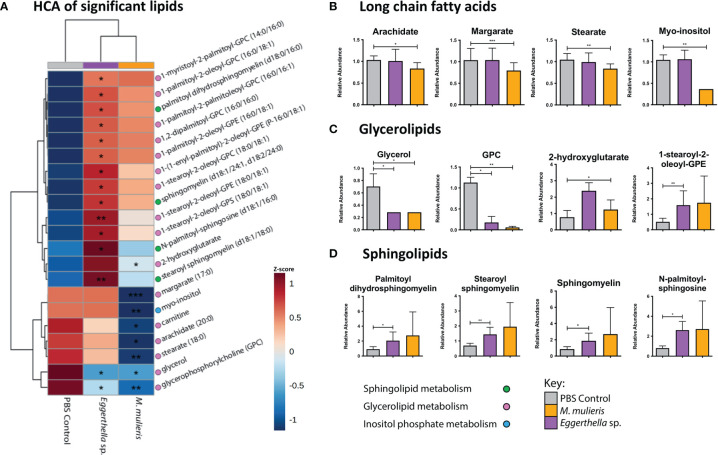
*Eggerthella* sp. significantly altered the abundance of more lipids than *M. mulieris.* **(A)** Hierarchical clustering analysis (HCA) of differentially abundant lipids (*p*<0.05) determined by Students t-tests with Welch’s correction of *Eggerthella* sp. MVA1 and *M. mulieris* UPII-28I infection compared to the PBS mock-infected control. HCA was performed using Euclidean distance measures and average linkage clustering algorithms. Relative abundance graphs of significant lipids classified into long-chain fatty acids **(B)**, glycerolipids **(C)** and sphingolipids **(D)**. The brackets after the lipids indicate how many carbons and how many double bonds there are in the structure of the lipid. The slash between numbers separates the information about the two hydrocarbon chains of the lipid, whilst the P- prefix indicates a neutral plasmalogen species and the d for sphingomyelins indicates a 1,3 dihydroxy chain. Significant differences between the bacteria and PBS mock-infected controls. *, *p*<0.05; **, *p*<0.01; ***, *p*<0.001.

Since both *M. mulieris* and *Eggerthella* sp. infections significantly modulated lipid metabolic pathways in culture supernatants, we identified the specific lipids with differential abundance (*p*<0.05) between bacterial infections compared to PBS mock-infected controls. We found 21 significantly altered lipids between both bacterial infections (**Figure 5A** and **Supplementary Figure 4**). These lipids can be classified into three categories of metabolism: sphingolipid metabolism, glycerolipid metabolism and inositol phosphate metabolism. Overall, *Eggerthella* sp. induced differential abundance of more lipids (16) than *M. mulieris* (7) and both significantly depleted the levels of glycerol (*p*=0.024 and *p*=0.0433, respectively) and glycerophosphorylcholine (GPC) *p*=0.0389 and *p*=0.00931, respectively) (**Figure 5C**). *Eggerthella* sp. infection mainly resulted in accumulation of glycerolipids and sphingolipids in contrast to *M. mulieris* which predominantly depleted long chain fatty acids; arachidate (*p*=0.0368), margarate (*p*=0.000919) and stearate (*p*=0.00504) (**Figure 5B**). Interestingly, the sphingolipids that were significantly altered by *Eggerthella* sp. were also elevated following *M. mulieris* infections but did not reach significance following infection with the latter species (**Figure 5D** and **Supplementary Figure 4**). Sphingolipids are closely linked to epithelial barrier function and inflammation (Hannun and Obeid, 2018; Harrison et al., 2018).

### *Eggerthella* sp. Infection Significantly Elevated Biogenic Amines and Other Metabolites Associated With BV Symptoms and Diagnosis, Whereas *M. mulieris* Infection Modulated Metabolites Related to Energy Metabolism and Oxidative Stress

**Figure 6:**
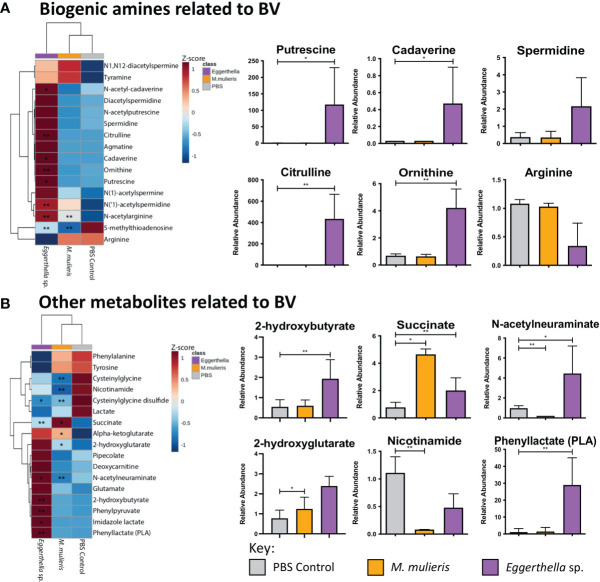
*Eggerthella* sp. significantly elevated the production of metabolites associated with biogenic amines and *M. mulieris* significantly altered metabolites related to energy metabolism. Hierarchical clustering analysis (HCA) and relative abundance of the BV-associated metabolites of *Eggerthella* sp. MVA1 and *M. mulieris* UPII-28I infection compared to the PBS mock-infected control: biogenic amines **(A)** and other metabolites related to BV symptoms **(B)**. HCA was performed using Euclidean distance measures and average linkage clustering algorithms. Statistical significance was calculated using Student’s t-tests with Welch’s correction compared to PBS mock-infected control; *, *p*<0.05; **, *p*<0.01.

**Figure 7:**
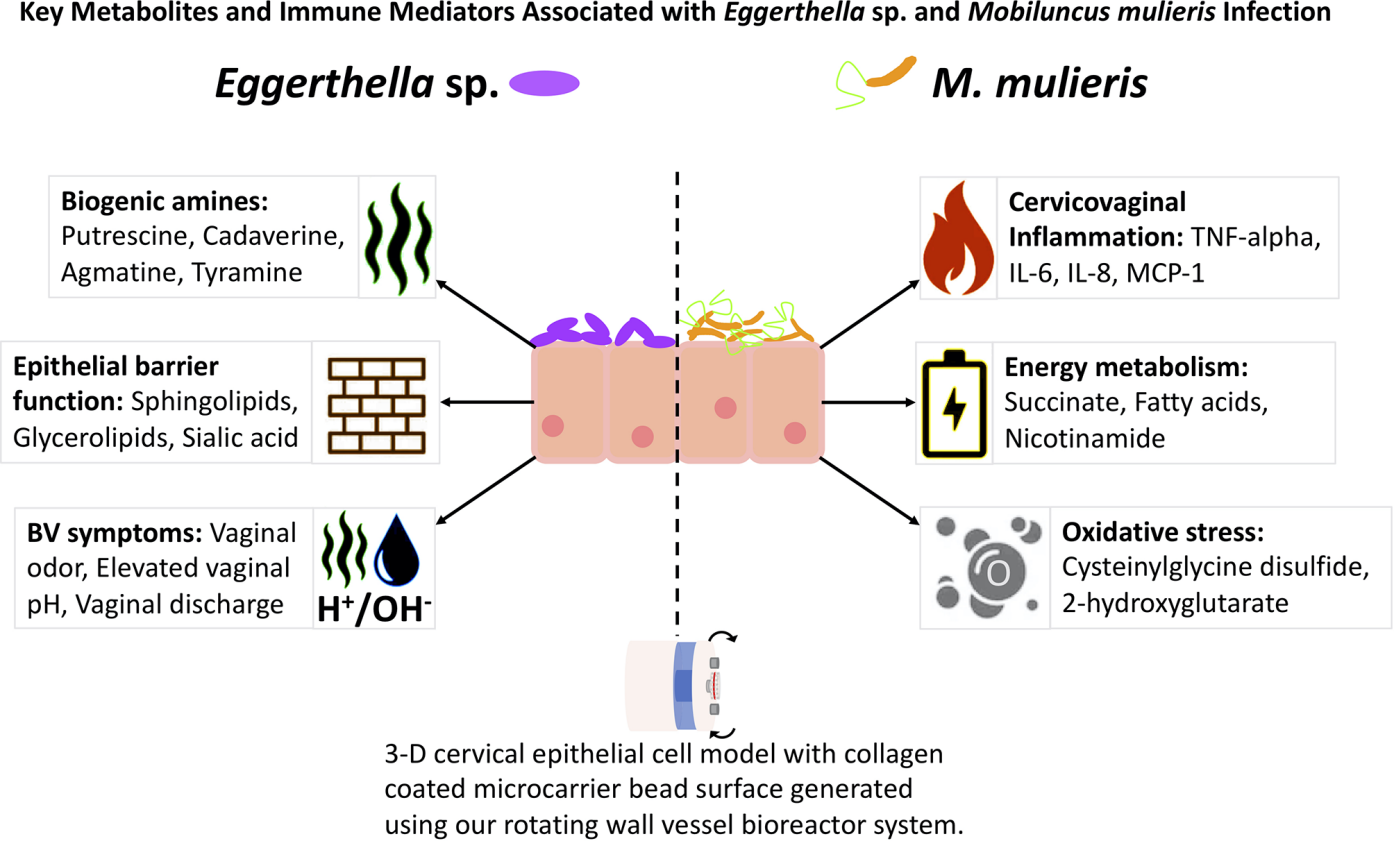
Comparisons of *Eggerthella* sp. and *M. mulieris* infections of 3-D cervical epithelial cell models. Supernatants from infections of 3-D cervical cell models with *Eggerthella* sp. MVA1 and *M. mulieris* UPII-28I were compared to PBS mock-infected controls in Bio-Plex and global metabolomics analyses. *M. mulieris* UPII-28I significantly increased inflammatory markers: IL-6 (*p*<0.0001), IL-8 (*p*<0.0001), MCP-1 (*p*<0.01) and TNF-α (*p*<0.01). Metabolites related to energy metabolism, such as nicotinamide (*p*=0.00429) and succinate (*p*=0.0335) were increased following *M. mulieris* UPII-28I infection but are relatively understudied in relation to BV. Furthermore, metabolites linked to oxidative stress, such as 2-hydroxyglutarate (*p*=0.0365), cysteinylglycine (*p*=0.00103) and cysteinylglycine disulfide (*p*=0.00238) were significantly altered by *M. mulieris* UPII-28I and are associated with inflammation. *Eggerthella* sp. MVA1 on the other hand significantly altered levels of the biogenic amines cadaverine (*p*=0.0373) and putrescine (*p*=0.0101) and elevated levels of sphingolipids, glycerolipids and sialic acid (*p*=0.0376), each of which are metabolites relating to the epithelial barrier function.

In clinical settings, BV is often diagnosed using the Amsel criteria (Amsel et al., 1983) which are based on the main symptoms of BV (vaginal pH, vaginal odor, vaginal discharge and the presence of clue cells). Thus, we determined whether infection with *Eggerthella* sp. or *M. mulieris* induced differential abundance of metabolites related to BV diagnosis in our 3-D human cervical cell models (**Figure 6**). We also evaluated the metabolites previously identified in cervicovaginal lavages collected from women with BV (Srinivasan et al., 2015). It is well established that biogenic amines are strongly associated with BV (Nelson et al., 2015) and linked to vaginal odor and elevated pH (Srinivasan et al., 2015; Borgogna et al., 2021). Cell culture supernatants from the 3-D cervical cell models infected with *Eggerthella* sp. significantly accumulated the biogenic amines cadaverine (*p*=0.0373) and putrescine (*p*=0.0101) and their precursors citrulline (*p*=0.00598) and ornithine (*p*=0.00669), as well as several other BV-related metabolites (**Figure 6A**, **Supplementary Figure 5**). In contrast, *M. mulieris* infections did not result in accumulation of any biogenic amines detected in our samples. *M. mulieris* infection significantly influenced metabolites related to energy metabolism: nicotinamide (*p*=0.00429) and succinate (*p*=0.0335); and oxidative stress: 2-hydroxyglutarate (*p*=0.0365) and cysteinylglycine (*p*=0.00103) (**Figure 6B** and **Supplementary Figure 5**). Intriguingly, relative abundance of one BV-related metabolite, N-acetylneuraminate (sialic acid), was significantly and differentially altered by both bacteria. Sialic acid was significantly increased by *Eggerthella* sp. (*p*=0.0376) and significantly decreased by *M. mulieris* (*p*=0.00886), which suggested that both bacteria possess sialidase activity. A potential reason why *M. mulieris* significantly decreased sialic acid could be due to it being able to catabolize sialic acid. Phenyllactate is a relatively understudied metabolite that was significantly upregulated by *Eggerthella* sp. infection (*p*=0.0028) and exhibited the largest fold change out of any metabolites detected in our data set (~1,150 fold). Overall, *Eggerthella* sp. infections significantly altered multiple metabolites related to BV symptoms, particularly biogenic amines and those linked to epithelial barrier function, such as sphingolipids and glycerolipids (Hannun and Obeid, 2008; Bittman, 2013; Jernigan et al., 2015). In contrast *M. mulieris* infections significantly increased metabolites related to energy metabolism and oxidative stress (**Figure 7**).

## Discussion

BV is characterized by colonization of the cervicovaginal epithelium by a diverse community of anaerobic bacteria. *M. mulieris* and bacterial species from the family *Eggerthellaceae* are relatively understudied bacteria compared to many of the other BV-associated microorganisms. In this study we aimed to examine how two vaginal isolates: *Eggerthella* sp. strain MVA1 and *M. mulieris* strain UPII-28I, influence the immunometabolic landscape in the context of the lower FRT, as well as the potential pathophysiological contributions of these species to BV.

In recent years, there has been a reclassification of members belonging to the *Eggerthellaceae* family leading to questions related to the contributions of specific family members to the BV state. Although genomic information is limited for *Eggerthella* sp. strain MVA1, there is a sequence read SRX655730 available in the NCBI Sequence Read Archive. The 16S rRNA gene was sequenced from isolate *Eggerthella* sp. MVA1 (JX103988) and shares 99% sequence identity with *Eggerthella lenta*, however the species was not assigned by the BEI repository. There is very little information about *E. lenta* in the FRT and the vaginal microbiome since it is predominantly a gut microbe. *E. lenta* is found at low abundance in the FRT and may be transferred to the vagina from the gastrointestinal tract (Priputnevich et al., 2021). Unfortunately, until the comparative genomic analysis is performed, we cannot classify a species for the strain *Eggerthella* sp. MVA1. The family *Eggerthellaceae* also contains *Coriobacteriales* bacterium DNF00809, previously classified as *Eggerthella* sp. type 1 by Srinivasan et al. (Srinivasan et al., 2016) before culture and whole genome sequencing of the species. Previous studies showed that this bacterium was present in 85-95% of women with BV compared to those without BV (Fredricks et al., 2005; Fredricks et al., 2007; Srinivasan et al., 2012; Shipitsyna et al., 2013). These previous studies have also shown that *E. lenta* is much less prevalent in the FRT than *Coriobacteriales* bacterium DNF00809, which is no longer classified as an *Eggerthella* species. Although beyond the scope of this study, an in-depth analysis of taxonomic classification of vaginal bacteria belonging to the family *Eggerthellaceae* should be further investigated and clear taxonomic nomenclature should be referenced for this family to reflect the complexity of its lineage and putative role in BV and the cervicovaginal environment. Our study provides data related to the immunometabolic contributions with one of these understudied vaginal strains from the family *Eggerthelleacae*.

It is important to investigate BV-associated bacteria in the cervical epithelium since disruption of the microbiota at this mucosal site can lead to PTB, increased STI acquisition and other gynecological sequalae (Brunham and Paavonen, 2020). BV can also lead to ascension of pathogenic bacteria to the upper FRT, therefore promoting endometritis and pelvic inflammatory disease (Eckert et al., 2002). To determine the individual immunometabolic contributions of *Eggerthella* sp. and *M. mulieris* within the cervical microenvironment, we utilized a bioreactor-derived 3-D cervical epithelial cell model (Barrila et al., 2010; Hjelm et al., 2010; Gardner and Herbst-Kralovetz, 2016) in combination with multiplex immunoassays and global untargeted metabolomic approaches to identify key metabolites and immune mediators, respectively, related to *M. mulieris* and *Eggerthella* sp. infections.

Organotypic 3-D human cervical epithelial cell models recapitulate many features of parental tissue that are not observed in monolayer cell culture models. Our advanced 3-D cell culture model exhibits physiologically relevant features, such as TLR expression, microvilli, intercellular junctional complexes and secretory material that could influence how BV-associated bacteria can colonize the model in a way similar to *in vivo* tissues (Gardner et al., 2020; Łaniewski and Herbst-Kralovetz, 2021; Salliss et al., 2021) These features provide a more accurate representation of the *in vivo* state which is more amenable to translational research efforts and studying host-microbe interactions; however, each model system has its strengths, weaknesses and utility (Hjelm et al., 2010; Radtke and Herbst-Kralovetz, 2012; Radtke et al., 2012; Herbst-Kralovetz et al., 2016). The close relation of 3-D human cervical epithelial cells to cervical tissue allows us to investigate how these pathogens can cause pathophysiological changes to the cervicovaginal microenvironment and epithelia. Using SEM, we demonstrated colonization of the 3-D cervical epithelial cell model by *Eggerthella* sp. and *M. mulieris* (**Figure 1**). We evaluated the cytotoxicity of each bacterium and determined that neither *Eggerthella* sp. nor *M. mulieris* infections induced significant cytotoxicity in cervical cells.

Inflammation is a driver of many disease processes including BV where it has been previously associated with PTB (Meis et al., 1995; Goldenberg et al., 2000; Romero et al., 2007). Our immune mediator analysis revealed that both *M. mulieris* and *Eggerthella* sp. induced a proinflammatory response in 3-D cervical epithelial cell models. *Eggerthella* sp. significantly increased IL-1α while *M. mulieris* significantly elevated IL-6, IL-8, MCP-1 and TNFα. *M. mulieris* has been linked to the significant elevation of IL-6, IL-8 and TNFα *in vitro* (Anahtar et al., 2015; Dude et al., 2020). Flagella, such as those expressed by *M. mulieris*, have been linked to activation of TLR5 (Hayashi et al., 2001; Dela Cruz et al., 2021) which leads to the stimulation of the NF-κB signaling pathway and, in consequence, upregulation of IL-6, IL-8 and TNFα (Nasu and Narahara, 2010). Intriguingly, upregulation of IL-6 and IL-8 as well as the NF-κB signaling pathway have been connected to PTB (Romero et al., 2014). Clinical studies showed that IL-6, IL-8 and TNFα are some of the most common cytokines associated with PTB (Murtha et al., 1998; Coleman et al., 2001; Romero et al., 2014; Ville and Rozenberg, 2018). However, there is no clear diagnostic marker for PTB and proinflammatory cytokine and chemokine profiles differing among women who deliver pre-term (Wei et al., 2010; Fettweis et al., 2019). In addition, elevation of proinflammatory markers including IL-6 and IL-8 are associated with HIV infection risk (Mlisana et al., 2012; Rodriguez Garcia et al., 2015). Genital inflammation can lead to an increased risk of STI acquisition, with the infected epithelia being damaged, allowing the pathogens that cause STIs access to deeper tissues (Passmore et al., 2016). Inflammation of cervical and vaginal tissues induces recruitment of immune cells to the lower FRT which facilitates the spread of HIV and other STIs (Masson et al., 2014; Anahtar et al., 2015; Masson et al., 2015; Fichorova et al., 2020).

Oxidative stress has been closely linked to inflammation (Reuter et al., 2010). Oxidative stress becomes damaging when there are a disproportional amount of reactive oxygen species (ROS), that overwhelm the antioxidants capacity of glutathione (Schafer and Buettner, 2001). High levels of ROS can induce cellular damage and promote many inflammatory states including cancer (Perwez Hussain and Harris, 2007; Reuter et al., 2010; Burton and Jauniaux, 2011) by recruiting inflammatory markers such as cytokines and chemokines and stimulating NF-κB signaling (Reuter et al., 2010). In this study, we found that *M. mulieris* significantly altered multiple metabolites associated with oxidative stress, including cysteinylglycine, cysteinylglycine disulfide and 2-hydroxyglutarate, whilst *Eggerthella* sp. significantly altered 2-hydroxybutyrate and cysteinylglycine disulfide. Depletion of cysteinylglycine and cysteinylglycine disulfide, two intermediates in the glutathione synthesis pathway, could either indicate increased glutathione biosynthesis or signify an increase in the levels of ROS. Disruption of redox balance due to the elevated levels of ROS has been linked to activation of cell signaling pathways including those responsible for the regulation of inflammatory cytokines and PTB (Schieber and Chandel, 2014; Moore et al., 2018). Notably, increase of ROS can lead to lipid peroxidation which can free lipids from cell membranes (Burton and Jauniaux, 2011; Moore et al., 2018). The lipid peroxidation and membrane damage by ROS might be a mechanistic link behind the increased concentrations of lipids following infections of 3-D cervical models with *Eggerthella* sp. and *M. mulieris*.

Through our global untargeted metabolomics analyses, we found that *Eggerthella* sp. significantly altered twice as many lipids as *M. mulieris*. Specifically, sphingolipids were significantly elevated exclusively by *Eggerthella* sp. We also found similar significantly altered glycerolipids and sphingolipids as those reported by Salliss et al., 2021 that were elevated following infection with another BV-related microorganism: *Megasphaera micronuciformis* (Salliss et al., 2021). Sphingolipids are components of eukaryotic cell membranes and have been related to proinflammatory signaling pathways and apoptosis (Kolter and Sandhoff, 2006; Hannun and Obeid, 2008; Hannun and Obeid, 2018). Our results demonstrate that *M. mulieris* induced higher abundance of sphingolipids and most glycerolipids relative to *Eggerthella* sp. and PBS mock-infected controls, although the levels did not reach significance. We hypothesize that this may be related to the observation that *M. mulieris* has been shown to increase membrane permeability in cervical epithelial cells grown on transwells inserts (Dude et al., 2020) potentially by freeing these membrane-associated lipids. Both bacteria induced extracellular accumulation of multiple lipids related to epithelial barrier function (Bittman, 2013; Jernigan et al., 2015). Considering these results, we hypothesize that *Eggerthella* sp. and *M. mulieris* may play a role in increasing membrane permeability, although significant cytotoxicity was not observed in our experiments. Unexpectedly, compared to other lipids long-chain fatty acids (LCFAs) were significantly depleted by *M. mulieris* infection. It is possible that the depletion of LCFAs could result from an ability of *M. mulieris* to catabolize these liberated LCFAs a as an energy source. Unfortunately, the genomic sequence of *M. mulieris* is not fully annotated, therefore it is unclear if this bacterial species synthesizes all proteins necessary to facilitate the catabolism of the LCFAs.

The epithelial barrier function and the physiological properties of the mucosal membranes lining the FRT are crucial in protecting the cervix from BV-associated bacteria (Rodriguez Garcia et al., 2015). One of the key pathophysiological changes during BV is disruption of the epithelial barrier function, which allows pathogenic bacteria to access deeper tissues and induce inflammation (Muzny et al., 2019). Sialic acid is the terminal sugar moiety on glycans of cell surface glycoproteins and mucins. The epithelium of the FRT is lined with highly glycosylated mucins which limit adhesion and colonization of pathogenic bacteria during BV (Linden et al., 2008; Barrila et al., 2010; Lewis and Lewis, 2012; Radtke et al., 2012). In addition, sialic acid residues can bind to pathogens and induce host cell signaling to generate an immune response (Macauley et al., 2014; Bhide and Colley, 2017). Significant alterations in the levels of sialic acid following bacterial infections indicates bacteria-mediated sialidase activity. Previous clinical studies have revealed elevated levels of sialidase and sialic acid in the cervicovaginal fluids of women with BV (Briselden et al., 1992; Moncla et al., 2015). Infection of 3-D cervical cell models with both *M. mulieris* and *Eggerthella* sp. significantly altered the levels of sialic acid, indicating that both species exert sialidase activity. As *M. mulieris* decreased the levels of extracellular sialic acid, we hypothesize that this species catabolizes sialic acid residues that are liberated from the cell surfaces (Culhane et al., 2006), similarly to other BV-associated bacterium *Gardnerella vaginalis* (Lewis et al., 2013). The sialidase activity of *M. mulieris* could play a role in PTB since the mucus plug created during pregnancy contains multiple mucins which could be degraded by sialidase, therefore allowing pathogenic bacteria to ascend to the uterus (Mcgregor et al., 1994; Lewis and Lewis, 2012; Smith-Dupont et al., 2017; Baker et al., 2018). Consequently, ascension of pathogenic bacteria into the upper FRT during pregnancy can lead to chorioamnionitis and PTB (Galinsky et al., 2013). *M. mulieris* and elevated levels of IL-8 have been previously associated with amniotic infection and PID (Hillier et al., 1988; Larsson et al., 1989; Hitti et al., 2001).

Sialidase activity has been noted as a potential diagnostic marker for BV (Briselden et al., 1992) along with several cervicovaginal metabolites, some of which are highlighted by Srinivasan et al., (2015). Amongst the metabolites associated with BV, biogenic amines are also considered key players in many aspects of BV pathogenesis (Nelson et al., 2015; Srinivasan et al., 2015; Borgogna et al., 2021). The Amsel criteria and Nugent scores are two methods to diagnose BV (Amsel et al., 1983; Nugent et al., 1991). Putrescine and cadaverine have been linked to decreased *in vitro* growth of *Lactobacillus* spp. and high Nugent scores in women with BV (Borgogna et al., 2021). Both putrescine and cadaverine are associated with increased vaginal pH, vaginal amine odor and vaginal discharge that manifest during BV (Srinivasan et al., 2012; Yeoman et al., 2013; Nelson et al., 2015). Through our metabolomics analysis we found that *Eggerthella* sp. significantly elevated both putrescine and cadaverine in the extracellular milieu, which corresponds with observations from Srinivasan *et al.* (Srinivasan et al., 2012). In contrast, *M. mulieris* did not elevate any biogenic amines in our current study. Previously, *M. mulieris* has been linked to elevated trimethylamine (Spiegel, 1991; Africa et al., 2014), however, this polyamine was not detected in our metabolomics analysis. Phenyllactate was found to be significantly elevated for *Eggerthella* sp. with the highest fold change (1150-fold) of all the metabolites measured. Although the role of this metabolite in the cervicovaginal microenvironment is still not clear, we have previously observed its accumulation following infections with other vaginal bacteria (Łaniewski and Herbst-Kralovetz, 2021; Salliss et al., 2021). The severity of BV has been associated with increased risk of HIV and other STI acquisition (Allsworth and Peipert, 2011); thus, contribution of *Eggerthella* spp. and *M. mulieris* to clinical symptoms of BV mechanistically links these species to poor health outcomes related to BV.

As with all experiments and biological models there are limitations (Herbst-Kralovetz et al., 2016). The 3-D cell culture model we have used is a robust tool that can provide mechanistic insights into host-microbe interactions in the cervical microenvironment. Our model, as with most human *in vitro* cell culture models, requires the use of pH-buffered medium; thus, it cannot mimic the acidic pH found in healthy women *in vivo* without impacting cellular viability However, the BVAB tested in this study thrive in a more neutral pH environment, which is a characteristic of our model (Barrila et al., 2010; Hjelm et al., 2010; Gardner and Herbst-Kralovetz, 2016). We also acknowledge that the bacterial strains used in this study may not represent the other closely related strains or species. As stated previously, the taxonomy of *Eggerthella* sp. MVA1 is still incomplete; therefore, we cannot generalize our findings to the other members of the *Eggerthellaceae* family. *Mobiluncus mulieris* is closely related to *Mobiluncus curtsii*; however, the two species are unique from each other in terms of physical characteristics and enzymatic activity. *M. curtsii* is smaller in size, can hydrolyze starch and hippurate and produce citrulline, ornithine and ammonia from arginine whilst *M. mulieris* cannot (Spiegel and Roberts, 1984). Future studies utilizing additional well-characterized bacterial isolates in mono- or polymicrobial infections are needed to better understand the individual contributions of these BVAB to poor gynecologic and obstetric outcomes.

Overall, we found *Eggerthella* sp. infections significantly altered multiple metabolites related to BV symptoms. These metabolites included the biogenic amines putrescine and cadaverine, as well as their precursors, and metabolites linked to epithelial barrier function, such as sphingolipids and glycerolipids (Ghosh et al., 1997; Bittman, 2013; Jernigan et al., 2015; Hannun and Obeid, 2018; Harrison et al., 2018; Heaver et al., 2018). *M. mulieris* infections significantly elevated multiple proinflammatory markers that are linked to PTB in addition to metabolites related to energy metabolism and oxidative stress (**Figure 7**). This study sheds light into the mechanisms that *Eggerthella* sp. and *M. mulieris* may utilize to promote BV. Our data suggests that *Eggerthella* sp. plays a key role in the production of biogenic amines, which contribute to the elevated vaginal pH and the amine odor, whilst *M. mulieris* potentially impacts the membrane permeability and induce proinflammatory immune responses. The increased concentration of lipids present in *M. mulieris* infection could also link into the increased immune response (Hannun and Obeid, 2018; Albeituni and Stiban, 2019; Sukocheva et al., 2020). The link to inflammation and the altered metabolic microenvironment fits into the hypothesis of Muzny et al. (2020) that proposes early colonizers establish biofilm and evade host defense responses whereas secondary colonizers mediate inflammation, an altered metabolic microenvironment and symptoms associated with BV. Based on our data, we propose that *M. mulieris* is functioning as a secondary colonizer in this hypothetical model of BV. In contrast, *Eggerthella* sp. while not inflammatory, exhibits metabolic activity consistent with our definition of a secondary colonizer, but may also participate in the early stages of biofilm formation. However, further *in vitro* studies investigating these microorganisms in polymicrobial settings in conjunction with longitudinal clinical studies are needed to elucidate microbe-microbe interactions and determine the role of these bacteria in the context of BV biofilms.

## Data Availability Statement

The original contributions presented in the study are included in the article/**Supplementary Material**, further inquiries can be directed to the corresponding author.

## Author Contributions

MH-K, PŁ, and JM conceived of the experimental design and interpretation of the data. JM conducted experimental infections of the 3-D cervical cell aggregates and the Bio-Plex analyses. Cell supernatants were sent to Metabolon, Inc. for global untargeted metabolomics analysis. RM carried out the cytotoxicity experiments as well as the analysis of the cytotoxicity data, Bio-Plex and metabolomics data. RM was also responsible for writing the first draft of the manuscript, drafting and editing the figures and revising the manuscript. JM obtained SEM images of infected 3-D human cervical cell models and assisted in the statistical analysis. MH-K, JM, and PŁ provided support and advice on writing, figures and tables, and also read and revised the manuscript. MH-K and PŁ provided guidance of the experimental and writing processes. MH-K supervised the research and provided funding acquisition, project administration and resources. All authors read, revised, and approved the final version of the manuscript.

## Funding

The funding for this study was supplied by the NIH National Cancer Institute (3P30CA023074-39S3) and the Flinn Foundation (2244) to MH-K.

## Conflict of Interest

The authors declare that the research was conducted in the absence of any commercial or financial relationships that could be construed as a potential conflict of interest.

## Publisher’s Note

All claims expressed in this article are solely those of the authors and do not necessarily represent those of their affiliated organizations, or those of the publisher, the editors and the reviewers. Any product that may be evaluated in this article, or claim that may be made by its manufacturer, is not guaranteed or endorsed by the publisher.
