# Supplementary material for: Immunometabolic Analysis of Mobiluncus mulieris and Eggerthella sp. Reveals Novel Insights Into Their Pathogenic Contributions to the Hallmarks of Bacterial Vaginosis
Source: Front Cell Infect Microbiol. 2021 Dec 23;11:759697. doi: 10.3389/fcimb.2021.759697 (PMC8733642; doi:10.3389/fcimb.2021.759697)
Supplement: Supplementary file 1 [file DataSheet_1.docx]

**Supplemental figures**

**Supplementary Figure 1: *M. mulieris* and *Eggerthella* sp. infection did not cause significant cytotoxicity of human cervical cells.** Monolayer cervical epithelial cell cultures infected with *M. mulieris* and *Eggerthella* sp. for 24 hours at 37°C under anaerobic contributions. Cytotoxicity was measured from cell culture supernatants using the CyQUANT LDH assay (Thermo Fisher Scientific) in accordance to the manufacturers protocol. Percentage LDH activity was measured by recording absorbance values at 490 nm and 680 nm and calculated according to the equation: $\frac{sample LDH activity}{lysed control LDH activity}\times100$.


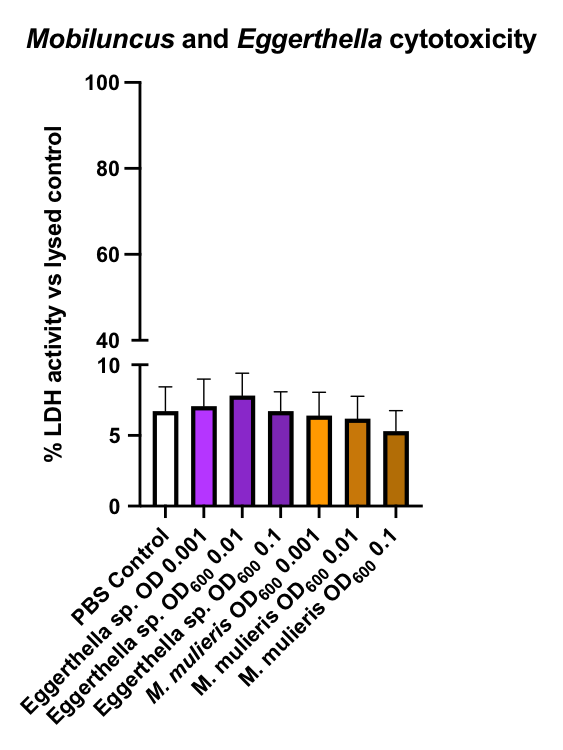


**Supplementary Figure 2: *M. mulieris* infections significantly elevated more pro-inflammatory mediators than *Eggerthella* sp. infections.** Immunoproteomics analysis was performed on cell culture supernatants from the 3-D cervical epithelial cell models infected with *M. mulieris* and *Eggerthella* sp. for 24 hours at 37°C under anaerobic conditions. The Bio-Plex analysis measured the concentrations of (**A**) Cytokines, (**B**) Chemokines and (**C**) Growth factors in these cell culture supernatants. Statistical significance was determined by one-way ANOVA and Bonferroni post-hoc multiple comparisons, *, *p*<0.05; **, *p*<0.01; ****, p*<0.001; ****, *p*<0.0001. Biological replicates were collected in triplicate for both *M. mulieris* and *Eggerthella* sp., with technical replicates being run in duplicate.

1. **Cytokines**

1. **Chemokines**

1. **Growth factors**

**Supplementary Table 1: *Eggerthella* sp. infection significantly altered more metabolites than *M. mulieris*.** Extracellular metabolites found to be significantly altered (*p*<0.05) by infection of 3-D cervical epithelial cells with *M. mulieris* or *Eggerthella* sp. were determined by using students t-test with Welch’s correction compared to PBS mock-infected controls. Fold changes represent relative abundance of metabolites from bacterial infections relative to PBS mock-infected controls. Metabolites are color-coded, indicating if they are significantly depleted (green) or significantly elevated (red) by each bacterial infection. Metabolites marked with a * indicate metabolites that are significantly (*p*<0.05) altered by both bacterial infections.

**Supplementary Figure 4: *Eggerthella* sp. infection significantly alters more lipids than *M. mulieris* infection.** Global untargeted metabolomic analysis of cell culture supernatants from 3-D cervical epithelial cells infected with *M. mulieris* or *Eggerthella* sp. revealed 21 significantly altered (*p*<0.05) lipids. The relative abundances of (**A**) glycerolipids, (**B**) sphingolipids and (**C**) inositol phosphate metabolism were predominantly modulated by *Eggerthella* sp. infection. Data was statistically analyzed using the Student’s t-test with Welch’s correction compared to PBS mock-infected controls. *, *p*<0.05; **, *p*<0.01; ****, p*<0.001.

1. **Glycerolipids**

1. **Sphingolipids**

1. **Inositol phosphate metabolism**

**Supplementary Figure 5: *Eggerthella* sp. infection significantly altered more biogenic amines than *M. mulieris*.** Cell culture supernatants from *M. mulieris* and *Eggerthella* sp. infections of 3-D cervical epithelial cell models were analyzed with global untargeted metabolomics. Metabolites relating to BV, specifically those relating to (**A**) biogenic amines and (**B**) other BV related metabolites were predominantly significantly altered by *Eggerthella* sp. compared to *M. mulieris*. Statistical significance was determined by Student’s t-tests with Welch’s correction compared to PBS mock-infected controls. *, *p*<0.05; **, *p*<0.01.

1. **Biogenic amine related metabolites**

1. **Other BV related metabolites**
